# Resolving Site-Specific Energy Levels of Small-Molecule Donor-Acceptor Heterostructures Close to Metal Contacts

**DOI:** 10.3390/nano11061618

**Published:** 2021-06-20

**Authors:** Amani Benhnia, Shinta Watanabe, Rouzhaji Tuerhong, Masato Nakaya, Jun Onoe, Jean-Pierre Bucher

**Affiliations:** 1 Institutde Physiqueet Chimiedes Matériaux de Strasbourg (IPCMS), Université de Strasbourg, CNRS, IPCMS UMR 7504, F-67034 Strasbourg, France; amani.benhnia@ipcms.unistra.fr (A.B.); ruzaji@gmail.com (R.T.); 2 Department of Energy Science and Engineering, Nagoya University, Furo-cho, Chikusa-ku, Nagoya 464-8603, Japan; s-watanabe@energy.nagoya-u.ac.jp (S.W.); m-nakaya@energy.nagoya-u.ac.jp (M.N.); j-onoe@energy.nagoya-u.ac.jp (J.O.)

**Keywords:** metal–organic interfaces, donor-acceptor molecular blends, scanning tunneling spectroscopy, self-assembly

## Abstract

The active material of optoelectronic devices must accommodate for contacts which serve to collect or inject the charge carriers. It is the purpose of this work to find out to which extent properties of organic optoelectronic layers change close to metal contacts compared to known properties of bulk materials. Bottom-up fabrication capabilities of model interfaces under ultrahigh vacuum and single-atom low temperature (LT)-STM spectroscopy with density functional theory (DFT) calculations are used to detect the spatial modifications of electronic states such as frontier-orbitals at interfaces. The system under consideration is made of a silver substrate covered with a blend of C_60_ and ZnPc molecules of a few monolayers. When C_60_ and ZnPc are separately adsorbed on Ag(111), they show distinct spectroscopic features in STM. However, when C_60_ is added to the ZnPc monolayer, it shows scanning tunneling spectra similar to ZnPc, revealing a strong interaction of C_60_ with the ZnPc induced by the substrate. DFT calculations on a model complex confirm the strong hybridization of C_60_ with ZnPc layer upon adsorption on Ag(111), thus highlighting the role of boundary layers where the donor-acceptor character is strongly perturbed. The calculation also reveals a significant charge transfer from the Ag to the complex that is likely responsible for a downward shift of the molecular LUMO in agreement with the experiment.

## 1. Introduction

Organic optoelectronics, in the form of organic photovoltaics (OPVs) and organic light-emitting (OLEDs) devices, has undergone a rapid development in recent years which is largely attributable to unique advantages such as lightweight, mechanical flexibility, large area, transparency, and low fabrication cost [1,2,3]. Much research effort has been directed at improving the performance of devices. To date, the power conversion efficiency of OPV devices has passed the 15% mark [4], whereas novel materials and device structures has allowed the external quantum efficiencies of OLEDs to exceed 30% [5].

The active layer of bulk-heterojunctions typically consists of electron-donor and electron-acceptor molecules, sandwiched between two charge selective ohmic contacts. It has been shown that the nature of ohmic transitions at contacts of organic solar cells has a significant impact on their performance [6], and a better definition of energy level alignment of molecule-molecule and molecule-contact interfaces are key elements for the improvement of performance [7]. Thus, new interlayer materials have been introduced for work function control and to favor selectivity and chemical compatibility [8,9,10]. In particular, the molecule–metal interface is involved when ultrathin silver as well as silver-doped Al layers and grids of silver or gold are used as semi-transparent electrodes in OPV in combination (or not) with metal-oxides [11,12,13,14,15]. The fact that, various light-trapping strategies in OPV consist in incorporating large area nanostructures such as Ag layers and nanostructures [16] brings even more relevance to this question.

Here, this issue is addressed in the framework of molecular blends of donor-acceptor molecules that are particularly relevant for applications. In this context, the measurement of electronic properties remains challenging because of the difficulty of interpretation of bulk measurements for small length-scales. To evaluate the incidence of metal contacts on ultrathin layers of molecules, an approach with atomic-scale control is necessary. Scanning probes have proven to be particularly powerful in monitoring and characterizing the growth of molecular structures on surfaces. Considering that in situ UHV conditions are necessary for controlled self-assembly of molecules, this task is ideally addressed by resorting to STM spectroscopy, provided that relatively small donor and acceptor molecules are considered.

In this study, emphasis is therefore put on metallophthalocyanines (MPc) in combination with C_60_ molecules. Whereas such systems have been analyzed quite in detail by means of various ensemble averaging techniques [10,11,17,18,19], the study of blends of molecules, by STM is rather scarce and has been mainly addressed from the growth point of view [20,21,22]. Here, we perform an in-depth spectroscopic study by high-resolution LT-STM of a ZnPc/C_60_ heterostructure in contact with a silver substrate. In addition, we aim at addressing the control of the molecular environment [23,24,25] and the charge transfer [26] which are important aspects of the preparation of optoelectronic devices.

## 2. Methods and Experimental Results

### 2.1. Experimental Methods

Experiments were performed in a UHV system with a base pressure *p* < 10^−10^ mbar, equipped with a low temperature scanning tunneling microscope (Modified Createc LT-STM). The Ag(111) single crystal was cleaned by repeated cycles of Ne^+^ ion bombardment followed by thermal annealing at 500 °C. The ZnPc and C_60_ molecules were evaporated from two Al_2_O_3_ crucible heated by means of a tantalum filament. Before the deposition, the ZnPc powder from Sigma-Aldrich (purity > 98%) is degassed for several hours in UHV. The ZnPc molecules are then deposited at a rate of 0.2 mL/min from the crucible held at a temperature of 180 °C on the silver substrate kept at room temperature. The C_60_ powder from Sigma-Aldrich (98% purity) has been sublimed at a crucible temperature of 240 °C. The sample is then transferred into the STM chamber. All STM/STS measurements have been performed at T = 77 K. Tungsten tips are flash annealed in UHV and conditioned by indentation into an Au substrate. STM images were acquired in a constant current mode, the bias voltage is applied to the sample with respect to the tip kept at a virtual ground. The dI/dV(V) signal was measured by a lock-in technique, superposing a rms modulation of 3.5 mV at a frequency of 600 Hz to the sample bias. Wide range dI/dV spectra are measured at a sample bias between −2.0 V and 2.0 V.

### 2.2. Experimental Results

The Ag (111) substrate is cleaned by repeated sputtering and annealing cycles. The molecular deposition is performed in an ultrahigh vacuum (UHV) chamber from two alumina crucibles, the first contains the ZnPc whereas the second one contains the C_60_, evaporation temperatures are T = 180 °C and T = 240 °C respectively. Both molecules are deposited onto the Ag substrate held at room temperatures. After deposition, the sample is transferred into the STM chamber held at a base pressure of *p* = 1 × 10^−11^ mbar where the STM experiments are performed at T = 77 K.

**Figure 1:**
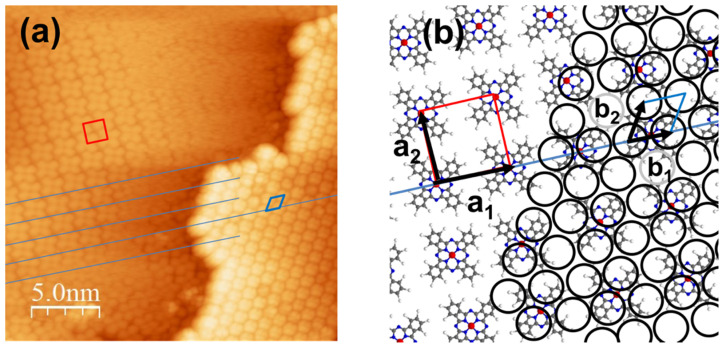
(**a**) STM topography of a C_60_ island grown on a ZnPc layer on Ag(111), simultaneously showing the molecular resolution of both lattices, see the red square and the blue diamond; the blue lines indicate rows of ZnPc molecules (0.6 nA, −0.4 V and T = 77 K). (**b**) Magnified schematic view corresponding to the STM topography, where the C_60_ lattice (circles) is superposed on the ZnPc lattice. As indicated **a_1_** and **b_1_** are colinear.

As an example, Figure 1a shows an STM image of a C_60_ island grown on a monolayer of ZnPc where both networks are well resolved. The relationship between the two structures is highlighted in the schematic view of Figure 1b. We anticipate that a two-monolayer-thick blend of donor and acceptor molecules in contact with a metal surface is sufficient to define the molecule–metal interface of a heterostructure. An incomplete C_60_ layer then has the advantage of presenting a different coordination of C_60_ molecules on top of the ZnPc layer, useful in the course of this study. Prior to growing the C_60_/ZnPc heterostructure on Ag(111), the growth mode of each molecular specie was investigated individually, see Figure S1. We found that the ZnPc forms a first uniform layer of flat laying molecules with a nearly square lattice, whereas the C_60_ exhibits a compact layer-by-layer growth on Ag(111).

**Figure 2:**
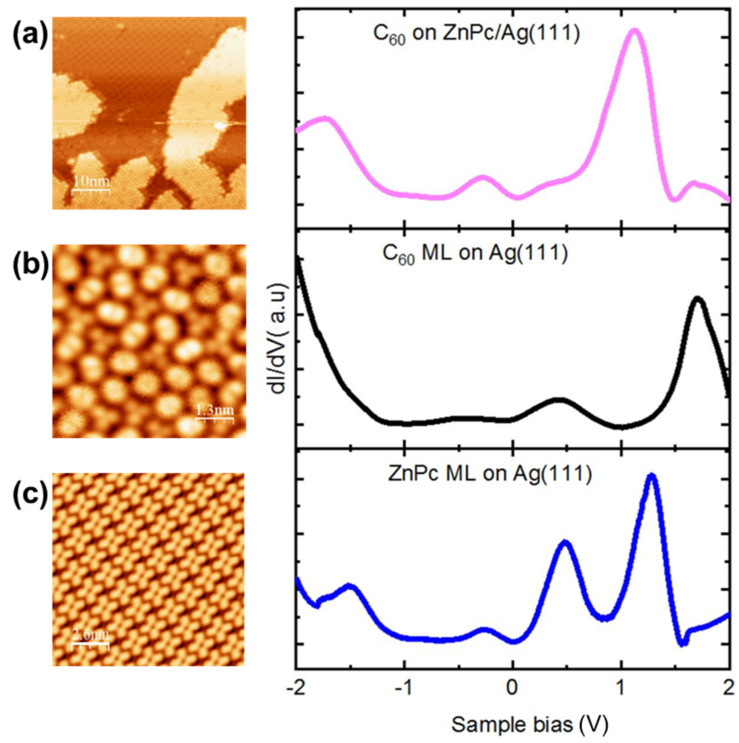
STM topography and differential conductance spectra for (**a**) C_60_ molecule islands grown on ZnPc/Ag(111). For comparison: (**b**) C_60_ molecules on Ag(111); the topograph shows contrasts of either the topmost hexagon ring or the 6:6 bond separating two adjacent hexagon rings of the C_60_ molecule. (**c**) Face-on ZnPc molecules on Ag(111). For the dI/dV spectroscopy, the feedback loop was opened at 0.7 nA and −0.3 V.

Combined photoemission and inverse photoemission works already found a dramatic effect of the metal contact on the HOMO-LUMO gap of C_60_ that drops from 3.3 eV for thick films to 2.3 eV for a C_60_ monolayer on Ag(111) [27,28]. At the same time, the information about ZnPc on substrates is scarce and no work has been done so far on silver. Here we find that two separate samples, one with a C_60_ monolayer on Ag(111) and another one with a ZnPc monolayer on Ag(111) show very distinct STS spectra (Figure 2b,c) leading to different HOMO levels and HOMO-LUMO gaps. In particular, the C_60_/Ag(111) spectrum of Figure 2b looks very much like the ones obtained in early studies [29,30,31,32] with same LUMO, LUMO+1 and HOMO. It is also noteworthy that spectra taken above C_60_ with different topographic contrasts (topmost hexagonal face or edge in Figure 2b) are identical. Surprisingly, when a C_60_ monolayer is deposited on top of a ZnPc layer, it is found that most resonances in the STS appear at same energies as for the ZnPc layer alone. This anomalous energy level alignment will be discussed in detail in the following.

As a matter of fact, the two peaks of C_60_/ZnPc/Ag(111) at −1.7 eV and +1.08 eV and the shoulder at 0.45 eV (Figure 1a) seem to come from the underlying ZnPc as can be observed from the ZnPc/Ag(111) spectrum of Figure 2c. Additionally, two small satellites of the main peak at 1.08 eV in Figure 1a seem to come from two remarkable features of C_60_/Ag(111) at +0.46 and +1.6 eV (compare Figure 2a,b). The small peak at −0.31 eV on C_60_/ZnPc/Ag(111) (Figure 1a) which is absent on C_60_/Ag(111) (Figure 2b) also stems from the ZnPc/Ag(111) (Figure 2c). The feature at −0.31 eV is not specific to ZnPc/Ag(111) and has been observed for MnPc/Ag(111) as well [33], showing strong evidence that this peak is substrate related. See also the case of FePc/Ag(111) [34].

The energy levels gathered from the observation are summarized in Table 1. The alignment for HOMO, LUMO, LUMO+1, and LUMO+2 suggests a strong hybridization of the ZnPc and C_60_ layers induced by the metal substrate. In addition, for all cases, a HOMO-LUMO gap comprised between 2.0 and 2.3 eV is found, not considering the small feature at −0.3 eV. For comparison, the corresponding molecular orbital energies for thick-layer, bulk heterostructures are shown as well [17]. It is found that the frontier orbitals of the adsorbed ZnPc are downward shifted by about 0.35 eV compared to the bulk heterostructure whereas the LUMO-level of the adsorbed C_60_ appears relatively unperturbed at +0.46 eV. The downward shifting of frontier orbitals compared to experimental bulk values is expected and is clearly an effect of the adsorption on the metal surface. See also the calculation in the next section. Regarding the C_60_/ZnPc blend on Ag(111), a weak shoulder at +0.4 eV and an intense peak at +1.1 eV are observed above the C_60_. The LUMO level can tentatively be assimilated to the intense peak at +1.1 eV, corresponding to the LUMO+1 of ZnPc on Ag (111). These results reveal an inversion of the usual donor-acceptor picture of ZnPc and C_60_ close to the contact.

It has been shown that small differences in the charge distribution at the bonding site of C_60_ can be responsible for as much as 0.6 eV increase in the gap when going from a silver to a gold substrate [30], demonstrating a large impact of the substrate. Although some analytical treatments have been applied to understand the sensitivity to the C_60_ environment [27,31] a better treatment of screening effects can only be addressed through a numerical approach. In order to improve our understanding of these effects, that are likely triggered by the on-surface adsorption of the molecular species, we made a detailed simulation of the adsorption of a C_60_/ZnPc complex on a silver substrate.

## 3. Model Calculation and Discussion

**Figure 3:**
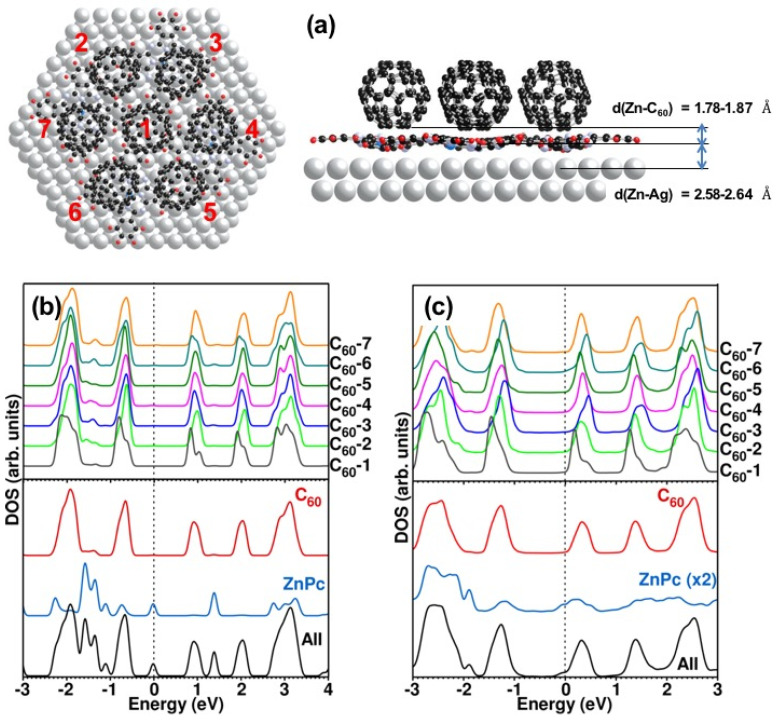
(**a**) 7 C_60_ + 4 ZnPc model complex adsorbed on Ag(111) slab used for the DFT calculation. The side view shows the structure of the complex after relaxation. LDOS on the a 7 C_60_ + 4 ZnPc model complex (**b**) in the gas phase (**c**) adsorbed on a Ag(111) slab. The LDOS spectra are numbered according to the position of in the complex, C_60_-1 being in the center whereas C_60_-2 to C_60_-7 are on the perimeter. The lower panels show total DOS on C_60_, ZnPc and all together. The energy axis represents (E − E_F_).

Due to the large number of atoms involved in the heterostructure, a model calculation on this system is very challenging. Therefore, to simulate the real system, we consider a C_60_ heptamer cluster lying on top of a ZnPc layer made of four ZnPc molecules. In the following, we call it the 7 C_60_ + 4 ZnPc complex (see Figure 3). The complex is designed to reproduce locally the relationship between the two lattices in accordance with Figure 1, see also Figure S1 and the SM for details. For the purpose of comparison, we considered both, a calculation of a free complex in the gas phase prior to deposition, and a calculation of the same complex deposited on a Ag(111) slab. The computational method is described in Appendix A.

All the structures have been relaxed prior to the DFT calculation to get the equilibrium configuration; see the side view of Figure 3a. The DFT calculation has been performed in the static configuration of the C_60_ molecules, where all the C_60_ molecules have the same face-on orientation of the C_60_ hexagon on the ZnPc layer. This assumption seems to be valid, at least judging from the experiment on the bulk material whose rotational motions are frozen below 90 K [35]. Since our experiments have been done at 77 K, we believe it is legitimate to assume a static configuration in your calculations. The calculation for the 6:6 bond orientation of adjacent six-membered rings of C_60_ and ZnPc were also tested, however, the energy of this system was slightly higher. 

Because we explore the most stable configuration of molecules in our model, we did not restrict the symmetry in our calculation. Considering the orientation of C_60_ molecules and the interaction between ZnPc and C_60_, a structure with high symmetry is not always the most stable. As a result, we do not expect the same local density of states (LDOS) between apparently symmetric configurations of C_60_ molecules on ZnPc as one would expect by considering the geometry prior to the optimization process.

Let us first discuss the LDOS of Figure 3b for the gas phase situation. The calculation shows distinct spectra on C_60_ and ZnPc (lower panel of Figure 3b) revealing only a weak hybridization between C_60_ and ZnPc, except perhaps for the states above 3 eV. The HOMO-LUMO gap of about 1.8 eV that can be drawn from the LDOS is only weakly underestimated compared to the experimental value of 2 eV. Remarkably, the LDOS on the different C_60_ of the heptamer show differences. This may be due to different positions of the C_60_ above the ZnPc, thus experiencing different interactions with the underlying ZnPc (see the charge density distribution). Another reason may be that each ZnPc molecule in the 4 ZnPc + 7 C_60_ complex is distorted to a different degree. The splitting of individual resonances has been observed in a previous calculation performed on C_60_ only [36].

It is instructive to compare this situation to the experimentally more relevant one of the complex adsorbed on Ag(111) substrate shown in Figure 3c. The LDOS now reveals a strong hybridization of C_60_ and ZnPc states, which is apparent in Figure 3c whereas the HOMO-LUMO gap of about 1.8 eV is unchanged and compares well with experimental results. In addition, a significant broadening of relevant HOMO and LUMO peaks is observed upon adsorption of the complex on the Ag(111) surface (compare Figure 3b,c). The strong interaction is also reflected in the deformation of the ZnPc molecules at the interface with the C_60_ molecules (Figure 3a). The important charge transfer from the silver substrate to the complex (see the analysis below) leads to a significant downwards shift of the Fermi level which is not in the middle of the HOMO-LUMO gap anymore but lies just below the LUMO (Figure 3c), meaning that the simulated system is a n-type semiconductor.

**Figure 4:**
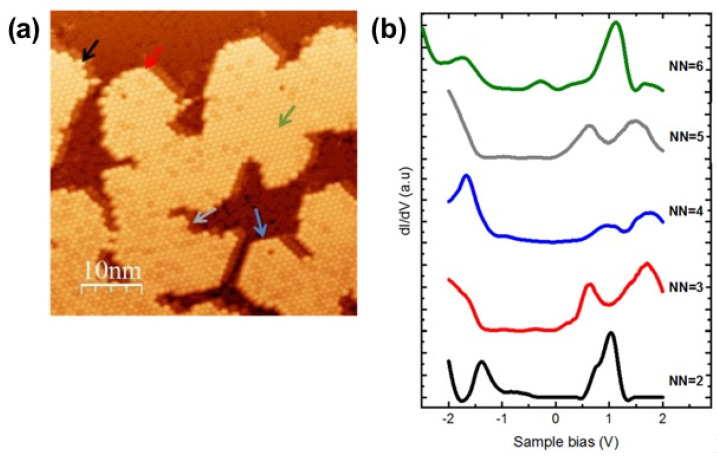
(**a**) STM topography of C_60_ islands grown on ZnPc/Ag(111). The arrows are highlighting the different coordination of C_60_ on top of the ZnPc layer. (**b**) Differential conductance spectra (dI/dV) of C_60_ molecule absorbed on a monolayer ZnPc on Ag (111) corresponding to different numbers of nearest neighbors in (**a**). STS were recorded for C_60_ molecules surrounded by 2 (black curve), 3 (red curve), 4 (blue curve), 5 (grey curve), and 6 (green curve) C_60_ molecule. Feedback loop was opened at 0.7 nA and −0.3 V.

Additionally, for both cases, free and adsorbed, we notice a difference in the LDOS of 6-fold coordinated C_60_ and the 3-fold coordinated C_60_ in the 4 ZnPc + 7 C_60_ complex model. To investigate this aspect further, we show in Figure 4 conductance spectra taken at various sites above a C_60_ island on ZnPc/Ag(111), corresponding to nearest neighbor (NN) C_60_ from 2 NN to 6 NN. The calculation correctly reproduces that the C_60_ with 6 neighbors (the central molecule of the heptamer cluster, Figure 3a) experiences a downward shift of both the HOMO and the LUMO compared to the C_60_ with 3 neighbors (outer molecules of the cluster, from C_60_-2 to C_60_-7 in Figure 3). As can be seen by comparing with the experimental conductance spectra of Figure 4, the trend is the same although the difference as a function of the C_60_ environment is not as large as observe in the experiments. In particular, we observe that the spectra for 3, 4, and 5 NN of C_60_ on ZnPc look very much like the C_60_ in direct contact with the Ag(111) substrate (see Figure 2b). This is clear from the HOMO-LUMO gap which is significantly larger than the LUMO-LUMO+1 gap in good agreement with the calculation of Figure 3c. It is not clear yet why the lower coordination in Figure 4 shows a dominant C_60_ feature, with a noticeable exception of the 2 NN case which looks more like the full coordination (6 NN). It must be emphasized that all spectra in Figure 4b have been tested for reproducibility on different parts of the C_60_ molecular islands. Unfortunately, it was not possible to fully probe these results by means of the calculation, due to the poor stability of clusters with small and intermediate NN numbers in the simulation. For example, the calculation with one C_60_ missing did not converge, i.e., the force acting on the C atoms of C_60_ did not fall below the convergence criteria (0.01 eV/Å) and the C_60_ kept on moving. This reflects the facts that voids are extremely difficult to simulate in finite systems (finite number of Ag atoms and ZnPc).

The Mulliken population analysis (Figure S2) provides additional information on the effect of the substrate. In the absence of the silver substrate the four ZnPc donate 1.0 e to the seven C_60_ molecules which is about 0.14 e per C_60_. In the presence of the silver substrate, however, the calculation shows that the silver donates to both, the ZnPc and the C_60_. We notice a large charge transfer from the silver (as much as 12 e) to the complex. This leads ultimately to about 0.5 e on each C_60_ molecule and 2 e on each of the ZnPc. These results confirm that the donor and acceptor character of ZnPc and C_60_ respectively is strongly perturbed in the vicinity of a silver electrode, since both types of molecules are now bearing a negative charge. Although the simulation gives the correct trend, the charge transfer is probably overestimated due to the finite size of the silver substrate in the simulation which underestimates the delocalization of free electrons in silver.

A strong reduction of the distance between the C_60_ and the ZnPc layer is observed upon adsorption, leading to a bending of the ZnPc. The average distance from the C_60_ to the ZnPc plane is reduced from 2.35 to 1.83 Å as shown in Figure 3. As a result, the adsorption of the complex on the Ag(111) substrate, produces a stronger interaction between ZnPc and C_60_ molecular layers that is also reflected in the significant broadening of the DOS peaks (Figure 3). In summary, both, the calculation and the experiment show a similar intriguing energy-level alignment for a C_60_ molecule on top of ZnPc/Ag(111). Our study furthermore highlights the presence of a Fermi-level that is located far from the mid-gap position, which is rather uncommon for an undoped organic semiconductor complex.

## 4. Conclusions

Our findings show that the proximity of contacts significantly alters the donor-acceptor picture originated from conventional organic electronics. When C_60_ is deposited on a ZnPc monolayer on a metal substrate, it shows similar STS (alignment of peaks) than ZnPc revealing a strong hybridization of the C_60_ with the ZnPc in the presence of the substrate. The calculation, including the silver substrate, clearly shows that the charge transfer from the silver contact cannot be neglected, leading to a downward shift of the molecular LUMO in good agreement with the experiment. The simulation confirms the strong hybridization of C_60_ layer with ZnPc layer upon adsorption on Ag(111). It is also found that the dI/dV spectra are very sensitive to the C_60_ nearest neighbor environment, thus underlining the importance of the boundary layers. Finally, one can anticipate that a significant charge transfer to the molecules is only expected for the first molecular layers of ZnPc and C_60_, those in the vicinity of the substrate. In actual optoelectronic devices, layers are several 10 nm thick, and the perturbation relaxes to a negligible value inside the layer. The findings are relevant for any optoelectronic system utilizing charge carrier injection or collection at contacts and interfaces and for photovoltaic devices in particular.

## Figures and Tables

**Table 1:** Experimental energy levels in eV.

	ZnPc/Ag(111)	C_60_/Ag(111)	C_60_/ZnPc/Ag(111)	ZnPc *	C_60_ *	7C_60_ + 4ZnPc/Ag(111) **
LUMO+2	-	+1.6	+1.6			+2.35
LUMO+1	+1.1	-	+1.1			+1.39
LUMO	+0.48	+0.46	+0.4	+0.82	+0.4	+0.32
SS	−0.3	-	−0.3			
HOMO	−1.6	−1.9	−1.7	−1.16	−1.9	−1.26

* values for bulk heterostructures from Park et al. [17]. ** Calculation (this work), the listed values correspond to peak values of the DOS.

## Data Availability

The data presented in this study are available on request from the corresponding author.

## Supplementary Materials

The following are available online at https://www.mdpi.com/article/10.3390/nano11061618/s1. Figure S1. Unit cell vectors corresponding to the ZnPc molecules. Inset: atomic resolution of the Ag(111); Figure S2. Mulliken charge distribution on the 4 ZnPc + 7 C_60_ complex in the gas phase (left) and adsorbed on the Ag(111) substrate (right).

## Appendix A

The model for the calculation is composed of four ZnPc and seven C_60_ molecules adsorbed on a two layer Ag(111) substrate, leading to a total of 968 atoms. A slab model with a 2.5 nm vacuum layer along the c-axis was adopted. The gas phase calculation was performed using the 4ZnPc + 7C_60_ model complex in a 3.608 × 3.608 × 2.5 nm^3^ vacuum box. The distance between the 4ZnPc + 7C_60_ complexes in neighboring cells is sufficient to prevent any artificial interaction between them. The geometry optimization and LDOS calculations were performed by DFT-D3 [37] method using the CASTEP [38], which is based on the ultrasoft pseudopotential plane-wave method [39]. Considering the calculation cost, the geometry optimization of the Ag(111) surface was performed first, and then used for the calculation of 4ZnPc + 7C_60_ complex on Ag(111). The exchange-correlation potential was considered within the generalized gradient approximation (GGA) proposed by Perdew, Burke, and Ernzerhof (PBE) [40]. The cutoff energy of the plane wave was 400 eV, and the reciprocal space integration was done using single gamma Monkhorst-Pack grids [41]. The geometry optimization was performed until the residual forces and stresses dropped below 0.01 eV/Å and 0.02 GPa, respectively.
