# Supplementary material for: Resolving Site-Specific Energy Levels of Small-Molecule Donor-Acceptor Heterostructures Close to Metal Contacts
Source: Nanomaterials (Basel). 2021 Jun 20;11(6):1618. doi: 10.3390/nano11061618 (PMC8234413; doi:10.3390/nano11061618)
Supplement: Supplementary file 1 [file nanomaterials-11-01618-s001.zip › nanomaterials-1254351-supplementary.pdf]

nanomaterials

MDPI

Supplementary Material

Resolving Site-Specific Energy Levels of Small-Molecule Donor-Acceptor Heterostructures Close to Metal Contacts

Amani Benhnia [1], Shinta Watanabe [2], Rouzhaji Tuerhong [1], Masato Nakaya [2], Jun Onoe [2] and Jean-Pierre Bucher [1,*]

[1] Université de Strasbourg, CNRS, IPCMS UMR 7504, F-67034 Strasbourg, France; amani.benhnia@ipcms.unistra.fr (A.B.); ruzaji@gmail.com (R.T.)

[2] Department of Energy Science and Engineering, Nagoya University, Furo-cho, Chikusa-ku, Nagoya 464-8603, Japan; s-watanabe@energy.nagoya-u.ac.jp (S.W.); m-nakaya@energy.nagoya-u.ac.jp (M.N.); j-onoe@energy.nagoya-u.ac.jp (J.O.)

* Correspondence: jean-pierre.bucher@ipcms.unistra.fr

1. Determination of the ZnPc Monolayer Lattice on Top of Ag(111)

The ZnPc molecules self-organize into highly ordered structures with a quadratic unit cell where each molecule is adsorbed face-on. From the STM-topography of Figure S1, we notice that the ZnPc molecule has four equivalent lobes, thus exhibiting a D_{4h} symmetry. Crystallographic $[1\bar{1}0]$ and $[11\bar{2}]$ directions of the silver substrate are labeled in the inset of Figure S1. The ZnPc lattice has the following parameters $b_1 = 1.50 \pm 0.07$ nm, $b_2 = 1.45 \pm 0.07$ nm with an angle $\beta = 92^{\circ} \pm 2^{\circ}$ between basis vectors.

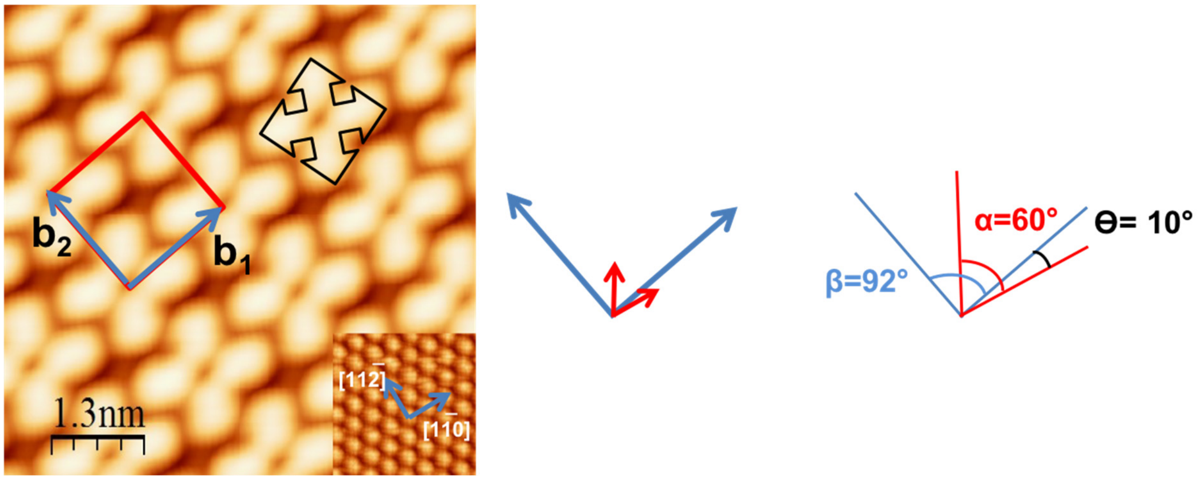

Figure S1. Unit cell vectors corresponding to the ZnPc molecules. Inset: atomic resolution of the Ag(111) substrate with crystallographic directions at the same scale as the ZnPc network.

Based on the STM images, we consider the primitive lattice vectors of the Ag(111) substrate to be $a_1 = a_2 = 0.286$ nm, with $\alpha = 60^{\circ}$. Here $\mathbf{a_1}$ is along $[1\bar{1}0]$ and the relation between the ZnPc and the Ag(111) lattice can therefore be defined by the following matrix:

$$\begin{bmatrix} 4.6 & 1.1 \\ -3.9 & 6.0 \end{bmatrix}$$

2. C_{60} Lattice in Relation to the ZnPc Molecular Network

From the STM topography it is found that the C_{60} molecules form a triangular lattice on top of the quadratic lattice of the ZnPc layer, the corresponding adsorption model is shown in Figure 1 of the article. The orientation and the atomic registry of the C_{60} lattice with respect to the ZnPc lattice can be determined and has the following parameters: $b_1 = 1.0$ nm, $b_2 = 1.0$ nm and $\beta = 60^{\circ}$. Based on the lattice of the ZnPc network, $a_1 = 1.50$ nm, a_2

= 1.45 nm, α = 90°, the primitive lattice vectors of the C_{60} superstructure can be expressed by means of the following matrix transformation:

$$\begin{bmatrix} 0.67 & 0 \\ 0.33 & 0.58 \end{bmatrix}$$

3. Mulliken Population Analysis

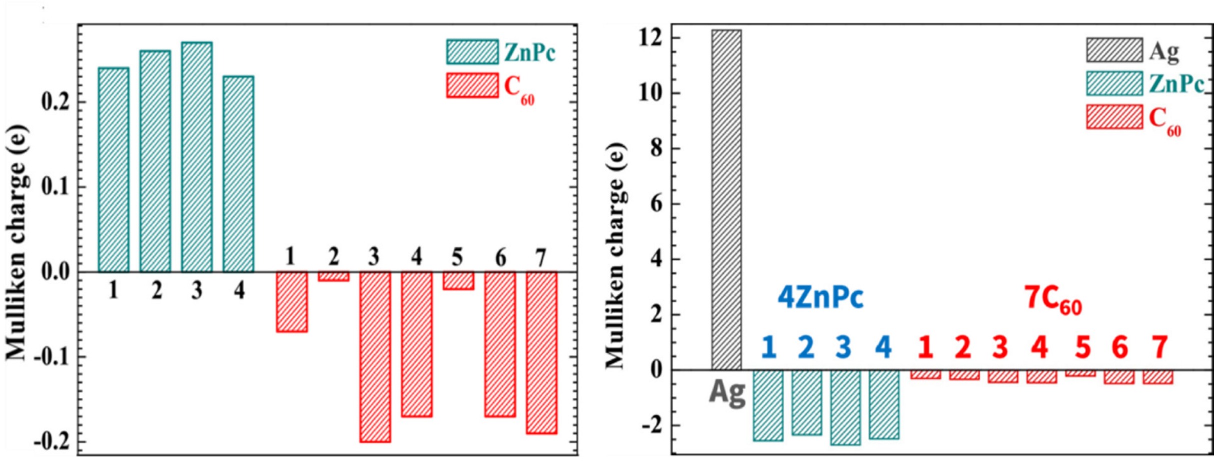

Figure S2. Mulliken charge distribution on the 4 ZnPc + 7 C_{60} complex in the gas phase (left) and adsorbed on the Ag(111) substrate (right).

The computation of Mulliken's population has been performed according to the original work of R. S. Mulliken, *J. Chem. Phys*. **1955**, 23, 1833.
